# Blocking EREG/GPX4 Sensitizes Head and Neck Cancer to Cetuximab through Ferroptosis Induction

**DOI:** 10.3390/cells12050733

**Published:** 2023-02-24

**Authors:** Aude Jehl, Ombline Conrad, Mickaël Burgy, Sophie Foppolo, Romain Vauchelles, Carole Ronzani, Nelly Etienne-Selloum, Marie-Pierre Chenard, Aurélien Danic, Thomas Dourlhes, Claire Thibault, Philippe Schultz, Monique Dontenwill, Sophie Martin

**Affiliations:** 1 Laboratory of Bioimaging and Pathology, University of Strasbourg, UMR7021 CNRS, 67401 Illkirch, France; 2 Department of Medical Oncology, Institute of Cancerology Strasbourg Europe, 67200 Strasbourg, France; 3 Laboratory of Design and Application of Bioactive Molecules, University of Strasbourg, UMR7199, CNRS, 67400 Illkirch, France; 4 Department of Pharmacy, Institute of Cancerology Strasbourg Europe, 67200 Strasbourg, France; 5 Department of Pathology, Strasbourg University Hospital, 67200 Strasbourg, France; 6 Department of Otolaryngology and Cervico-Facial Surgery, Strasbourg University Hospital, 67200 Strasbourg, France

**Keywords:** head and neck cancers, EREG, ferroptosis, tumoroid, biomarkers, metabolism, autophagy

## Abstract

(1) Background: Epiregulin (EREG) is a ligand of EGFR and ErB4 involved in the development and the progression of various cancers including head and neck squamous cell carcinoma (HNSCC). Its overexpression in HNSCC is correlated with short overall survival and progression-free survival but predictive of tumors responding to anti-EGFR therapies. Besides tumor cells, macrophages and cancer-associated fibroblasts shed EREG in the tumor microenvironment to support tumor progression and to promote therapy resistance. Although EREG seems to be an interesting therapeutic target, no study has been conducted so far on the consequences of EREG invalidation regarding the behavior and response of HNSCC to anti-EGFR therapies and, more specifically, to cetuximab (CTX); (2) Methods: EREG was silenced in various HNSCC cell lines. The resulting phenotype (growth, clonogenic survival, apoptosis, metabolism, ferroptosis) was assessed in the absence or presence of CTX. The data were confirmed in patient-derived tumoroids; (3) Results: Here, we show that EREG invalidation sensitizes cells to CTX. This is illustrated by the reduction in cell survival, the alteration of cell metabolism associated with mitochondrial dysfunction and the initiation of ferroptosis characterized by lipid peroxidation, iron accumulation and the loss of GPX4. Combining ferroptosis inducers (RSL3 and metformin) with CTX drastically reduces the survival of HNSCC cells but also HNSCC patient-derived tumoroids; (4) Conclusions: The loss of EREG might be considered in clinical settings as a predictive biomarker for patients that might undergo ferroptosis in response to CTX and that might benefit the most from the combination of ferroptosis inducers and CTX.

## 1. Introduction

With nearly 932,000 new cases and 450,000 deaths in 2018 worldwide, head and neck cancers, and, more particularly, head and neck squamous cell carcinoma (HNSCC), rank sixth among the most frequently observed cancers in the world [1]. Locally advanced HNSCC (LA-HNSCC, stage III/IV) represents 70% of patients at diagnosis. They require primary surgery followed by adjuvant (chemo)-radiotherapy or definitive chemoradiotherapy including cetuximab (CTX) [2,3]. The chimeric antibody CTX targets the epidermal growth factor receptor (EGFR), which happens to be overexpressed in more than 90% of HNSCC. CTX prevents ligand binding and dimerization with other HER family members [4,5]. Once bound, CTX blocks EGFR phosphorylation and signal transduction and promotes EGFR internalization, thus turning down the oncogenic EGFR signaling [6,7]. Unfortunately, some patients do not benefit from CTX treatment, and others show recurrences soon after the end of the treatment. Intrinsic or therapeutically acquired resistance to CTX is extensively studied to understand the mechanisms involved. We have recently shown the involvement of the caveolin-1/epiregulin/YAP axis in the resistance to CTX and irradiation therapy [8].

Epiregulin (EREG), encoded by the EREG gene located on chromosome 4q13.3, belongs to the ErbB family of ligands. The 162 amino acids transmembrane proform of EREG is proteolytically cleaved by ADAM17 [9] to release a soluble form of 46 amino acids [10]. EREG shares 24–50% of its sequence with those of other members of the EGF family [10]. EREG binds to EGFR (extracellular domain I and III that partially overlaps the EGF binding site [11]) and ErbB4. It stimulates homodimers of EGFR and ErbB4 in addition to heterodimers of ErbB2 and ErbB3, leading to the activation and the transduction of downstream signaling pathways [12]. In contrast to EGF, EREG leads to complete EGFR recycling and not to lysosomal degradation [13]. EREG induces less stable EGFR dimers than EGF, but unexpectedly, this weakened dimerization elicits more sustained EGFR signaling than EGF [14]. Low or non-existent in most human tissues, the elevation of EREG expression is observed in the early stages of cancer development, in which EREG induces epithelial-mesenchymal transition [15]. EREG is a transcriptional target of the oncogenic KRAS and is also overexpressed in cells with an oncogenic mutation of EGFR and BRAF [16]. EREG also promotes tumorigenicity, metastasis, drug resistance and cell plasticity and modulates the tumor microenvironment and metabolism [17]. The increased expression of EREG is associated with short overall survival in patients with HNSCC/OSCC [11,15,18,19,20]. Elevated levels of EREG appear to be a potential predictive biomarker of anti-EGFR therapies in several cancer types including HNSCC [21]. We recently reported that the overexpression of caveolin-1 in HNSCC is associated with the total loss of EREG as well as the oncogenic addiction to EGFR. Silencing EREG activates the YAP/TAZ pathway, which enables cells to resist CTX/radiotherapy. The resistance of HNSCC cells to therapy is linked to the protection of the mitochondria and drives the recurrence of caveolin-1-expressing HNSCC tumors [8].

How the loss of EREG affects the cellular metabolism and how it might influence the response to anti-EGFR therapies is only sparsely documented in HNSCC. Here, we aimed to clarify this point and highlighted that the loss of EREG sensitizes cells to CTX through the induction of ferroptosis. Our study reveals the glutathione metabolism as a targetable vulnerability of HNSCC that should be exploited in CTX-based therapies.

## 2. Materials and Methods

### 2.1. Cell Culture, Transfection and Drugs

The CAL27, CAL33 and SCC9 cell lines were purchased from the ATCC^®^ and DSMZ (authenticated by STR profiling). All cell lines tested negative for mycoplasma contamination. CAL27 and CAL33 were grown in DMEM (PAN Biotech, Aidenbach, Germany) supplemented with 2 mM ultra-glutamine, 0.5 mM sodium pyruvate and 10% heat-inactivated FBS (Gibco, Dutscher SAS, Brumath, France). SCC9 were grown in DMEM-F12 (PAN Biotech) supplemented with 2.5 mM ultra-glutamine, 15 mM HEPES, 400 ng/mL hydrocortisone (Sigma-Aldrich, St Quentin Fallavier, France) and 10% FBS (Gibco). EREG expression was downregulated by transfecting the cell lines with 25 nM siRNA_EREG_ (Human EREG and the respective control siRNA_Ctrl_, SMARTPool from Dharmacon) using Lipofectamine 2000^TM^ (Invitrogen, Thermo Fischer Scientific, Illkirch, France). Efficient EREG silencing was determined by Western blot. When indicated, cells were treated with solvent, 30 nM of CTX (Erbitux™, 5 mg/mL, Merck, ICANS, Strasbourg, France), 5 µM RSL3 (MedChemExpress, Clinisciences, Nanterre, France) or 1 mM metformin (MedChemExpress) alone or in combination with CTX.

### 2.2. Sphere Evasion Assay

After the treatments, 500,000 cells were resuspended in 1 mL of regular culture medium supplemented with 20% methylcellulose. Spheroids were formed using the hanging drop culture method. Drops of 20 µL cell suspension were placed onto the lids of 60 mm dishes, which were inverted over the dishes. The dishes were cultured in humidified chambers for 48 h to allow the formation of round aggregates. The spheroids were harvested and seeded in plastic 24-well plates (6 spheres/well) for an additional 24 h to allow for the evasion of cells from the attached spheres. Pictures were taken using the Evos XI Core microscope (AMG, Thermo Fischer Scientific, Illkirch, France), with 10× magnification. The results were expressed, in pixels, as the evasion area of the cells relative to the area of the attached sphere (total area − area of the sphere), determined using ImageJ (https://imagej.nih.gov, 1.53t).

### 2.3. IncuCyte^®^ Assay

After transfection, the cells were seeded (4000 for CAL33 and 8000 for CAL27 and SCC9 cells/200 µL/well) in 96-well plates. The plates were placed at 37 °C, and the confluence, growth, cell health and morphology were monitored for 164 h/7 days. The percentage of confluence was determined using the IncuCyte^®^ analysis software after normalization to day 0 (Essen BioScience, Sartorius, Goettingen, Germany).

### 2.4. Clonogenic Survival Assay

A total of 72 h after the transfection and/or an additional 48 h of treatment with the solvent, 30 nM CTX, 5 µM RSL3, 1 mM metformin or co-treatments, the cells were seeded (500 for CAL27 and SCC9 and 1000 for CAL33 cells/2 mL/well) in 6-well plates and allowed to grow for 10 days. The cells were colored with crystal violet at 0.1% (Sigma-Aldrich, St Quentin Fallavier, France). The colonies were counted to determine the plating efficiency (PE) and the surviving fraction (SF). PE = number of surviving cells/number of cells plated. SF = PE of the experimental group/PE of the control group.

### 2.5. Metabolic Assays

After the treatment, 20,000 cells were plated in a Seahorse XF Cell Culture microplate in XF growth medium (non-buffered DMEM containing 10 mM glucose, 4 mM L-glutamine and 2 mM sodium pyruvate). The OCR (oxygen consumption rate), ECAR (extracellular acidification rate) and ATP consumption were measured using the ATP rate assay procedure under basal conditions and in response to 1.5 μM oligomycin and 0.5 µM rotenone/antimycin A with the XFp Extracellular Flux Analyzer (Seahorse Bioscience, Agilent, Les Ulis, France). The metabolic profiles were analyzed using the online Seahorse analytics platform.

### 2.6. Western Blot

A total of 72 h after the transfection and/or an additional 48 h of treatment with the solvent, 30 nM CTX, 5 µM RSL3, 1 mM metformin or co-treatments, the cells were lysed with the lysis buffer (1% Triton, 100 nM NaF, 10 mM Na_4_O_7_P_2_, 1 mM Na3VO4, protease inhibitor cocktail (Roche, Meylan, France) in PBS) for 30 min at 4 °C and then sonicated. The supernatant was recovered by centrifugation at 20,000× *g* for 10 min at 4 °C. In total, 5 to 20 μg of proteins were separated on a 4–20% TGX-denaturing polyacrylamide gel (SDS-PAGE Bio-Rad Marnes-La-Coquette, France) and transferred to polyvinylidene difluoride (PVDF) membrane (Amersham, Sigma-Aldrich, St. Quentin Fallavier, France). After 1 h of blocking at room temperature, the membranes were probed with appropriate primary antibodies (see Supplementary Table S1) overnight at 4 °C. The membranes were subsequently incubated with anti-rabbit or anti-mouse antibodies conjugated to horseradish peroxidase (Promega, Charbonnieres les-Bains, France), developed using chemoluminescence (ECL, Bio-Rad, Marnes-La-Coquette, France) and visualized with an Las4000 image analyzer (GE Healthcare, Tremblay-en-France France). The quantification of non-saturated images was carried out using ImageJ software (National Institutes of Health, Bethesda, MD, USA). GAPDH was used as the loading control. The results were expressed as histograms representing the mean ± SEM of the ratios protein/GAPDH normalized against the controls.

### 2.7. Quantification of Intracellular Fe^2+^ Accumulation

A total of 72 h after the transfection and/or an additional 48 h of treatment with 30 nM CTX, the cells were seeded at 20,000 cells for CAL27 and CAL33 and at 15,000 cells for SCC9 for 24 h in 96-well plates with opaque walls. The intracellular accumulation of Fe^2+^ was determined using the intracellular probe FerroOrange at 1 µM for 30 min (Dojindo, TebuBio, LePerray en Yvelines, France). The fluorescence intensity was measured with a Varioskan LUX (Thermo Scientific, Illkirch, France) plate reader. In parallel, the cells were seeded at 30,000 cells for CAL27 and at 20,000 cells for CAL33 and SCC9 in an eight-well LabTek for imaging with an LEICA TCS SPE II confocal microscope (Leica Microsystems SA, Nanterre Cedex, France), with a ×20 magnification objective, and analyzed with ImageJ software.

### 2.8. Detection of the Accumulation of Lipid Peroxides

A total of 72 h after the transfection and/or an additional 48 h of treatment with 30 nM CTX, the cells were seeded for 24 h at 30,000 for CAL27 and at 20,000 for CAL33 and SCC9 in an eight-well LabTek. The accumulation of lipid peroxides was determined using the Liperfluo kit at 1 µM for 30 min (Dojindo, China). The cells were also seeded into LabTek wells for imaging with an LEICA TCS SPE II confocal microscope (Leica Microsystems SA, Nanterre Cedex, France), with a ×20 magnification objective, and analyzed with ImageJ software (https://imagej.nih.gov, 1.53t).

### 2.9. Tumoroids Culture

The study was approved by the Scientific Committee of the tumor bank and the Department of Pathology of the CHU Strasbourg-Hautepierre (France). The patients have signed an informed consent form. Tumor extractions were carried out in the Department of Cervico-Facial Surgery of the CHU Strasbourg-Hautepierre (France). The resected pieces were histologically diagnosed. The tumoroids were extracted from head and neck cancer surgical resection following the protocol developed by Driehus et al. [22] and cultured in advanced DMEM/F12 supplemented with GlutaMax, Penicilin/streptomycin, 10 mM HEPES, 10 µM Y-27632 (Euromedex, Souffelweyersheim, France), 0.5 µg/mL Capsofungin (Sigma), 1× B27 supplement (Thermo Fisher Scientific), 1.25 mM N-acetyl-L-cysteine (Sigma-Aldrich), 10 mM Nicotinamide (Sigma-Aldrich), 500 nM A83-01 (Sigma-Aldrich), 0.3 µM CHIR99021 (Sigma-Aldrich), 50 ng/mL human EGF (PeproTech, Thermo Scientific, Illkirch, France), 10 ng/mL human FGF10 (PeproTech), 5 ng/mL human FGF2 (PeproTech), 1 µM Prostaglandin E2 (Bio-techne, R&D Systems, Noyal Châtillon sur Seiche, France) and 1 µM Forskolin (Bio-techne), 4% (*col*/*col*) RSPO3-Fc fusion protein conditioned medium (ImmunoPrecise, IPATherapeutics, Utrecht, Netherlands) and 4% (vol/vol) Noggin-Fc fusion protein conditioned medium (ImmunoPrecise). Quality control of the tumoroids was performed. The tumoroids were plated at 2500 cells/10 µL of 70% Cultrex UltiMatrix reduced growth factor basement membrane Extract (R&D Systems, Noyal Châtillon sur Seiche, France) in 24-well plates. The tumoroids were treated with the solvent, 30 nM CTX, 5 µM RSL3, 1 mM metformin or co-treatments 7 days after plating for an additional 10 days. The cell viability was assessed after the exposure of the cells to trypan blue (Bio-Rad) and reading via a TC20 Automated Cell Counter (Bio-Rad). Moreover, this culture was monitored by imaging at ×4 and ×20 magnification via an Evos XI Core microscope (AMG).

### 2.10. Immunohistochemistry on Tumoroids

Following the recovery, the tumoroids were fixed in PFA 4% for 20 min and washed in PBS. After a 15 min permeabilization step in PBS/0.1% Tween-20 and a 60 min blocking step in PBS/0.1% Triton X-100/2% BSA/5% NGS, the tumoroids were incubated overnight at 4 °C with primary antibodies (Rabbit anti-EREG, #CSB-PA007779NA01HU, Cusabio Technology, dilution 1/300; Mouse anti-Caveolin-1, #66067-1-lg, Proteintech, dilution 1/1000). After washing in PBS/0.1% Triton X-100/0.2% BSA, the cells were incubated for 3 h at room temperature with appropriate secondary antibodies (Life Technologies; dilution 1/500) and DAPI (#D9542; Sigma-Aldrich, St Quentin Fallavier, France; 1 µg/mL). After washing twice in PBS/0.1% Triton X-100/0.2% BSA and twice in PBS, the slides were mounted using FUnGI medium (50% (*v*/*v*) glycerol, 9.4% (*v*/*v*) dH_2_O, 10.6 mM Tris base, 1.1 mM EDTA, 2.5 M fructose and 2.5 M urea). Images were acquired using an LEICA TCS SPE II confocal microscope (Leica Microsystems SA, Nanterre Cedex, France), with a 20× magnification objective, and analyzed with ImageJ software (https://imagej.nih.gov, version 1.53r, access on 3 May 2021) or Imaris software (Imaris x64 9.3.1—22 May 2019).

### 2.11. Statistical Analysis

Quantitative variables are presented as their mean and standard deviations and were compared to univariate analyses with a Student’s *t*-test if they followed a Gaussian distribution (Shapiro–Wilk tests were used to assess the Gaussian distribution) or with a Wilcoxon’s rank test if they followed a non-Gaussian distribution.

## 3. Results

### 3.1. Silencing EREG Prevents Survival and Growth and Sensitizes to CTX

**Figure 1:**
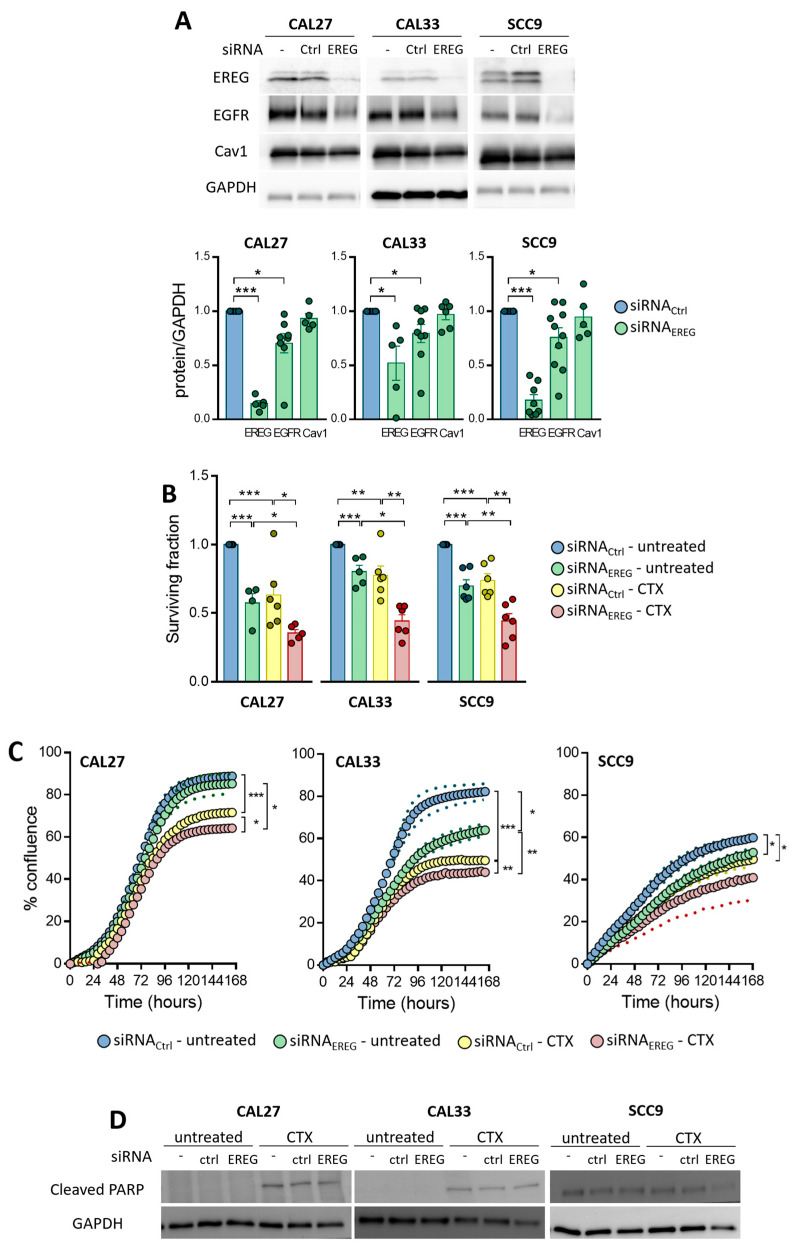
(**A**) Expression of EREG, EGFR, CAV1 and GAPDH was determined by Western blot in CAL27, CAL33 and SCC9 cells untransfected and transfected with siRNA_Ctrl_ or siRNA_EREG_. Histograms represent the mean ± SEM (CAL27 and CAL33 *n* = 6–11 and SCC9 *n* = 6–13, with * *p* < 0.05 and *** *p* < 0.001) of the protein expression in siRNA_Ctrl_- or siRNA_EREG-_transfected cells normalized with GAPDH. (**B**) Histograms show the surviving fraction of CAL27, CAL33 and SCC9 cells transfected with siRNA_Ctrl_ or siRNA_EREG_ and treated with solvent or 30 nM CTX. Data represent the mean ± SEM surviving fraction at day 8 post-transfection and post-treatment (*n* = 4 with * *p* < 0.05, ** *p* < 0.01 and *** *p* < 0.0001). (**C**) Curves show the percentage of confluence after normalization to day 0 of CAL27, CAL33 and SCC9 cells transfected with siRNA_Ctrl_ or siRNA _EREG_ and treated with solvent or 30 nM CTX. Data are represented as the mean ± SEM (small dots) of confluence at 168 h post-transfection and post-treatment (CAL27 and CAL33 *n* = 4–5 and SCC9 *n* = 6–5 with * *p* < 0.05, ** *p* < 0.01 and *** *p* < 0.001). (**D**) Expression of cleaved PARP and GAPDH was determined by Western blot in CAL27, CAL33 and SCC9 cells transfected with siRNA_Ctrl_ or siRNA_EREG_ and treated with solvent or CTX (30 nM).

We recently reported that decreased EREG expression conferred Cav1-overexpressing cells resistance to CTX/radiotherapy [8]. We postulated that it was the result of a decrease in EREG-driven oncogenic addiction to EGFR. To go further, EREG was silenced in a panel of three basal-like HNSCC cell lines using siRNA. The basal expression of Cav1, EREG and EGFR was not altered by the transfection of siRNA_ctrl_. Silencing EREG does not alter Cav1 expression and exhibits a significant reduction in EGFR, which is in contrast with the molecular alterations observed previously (Figure 1A). EREG-silenced cells (siRNA_EREG_) show reduced clonogenic survival (Figure 1B). Although CTX significantly reduces the survival of control (siRNA_ctrl_-transfected) cells, its effect is even more pronounced in EREG-silenced cells (Figure 1B; with 33 ± 10% and 51 ± 5% inhibition by CTX for siRNA_ctrl_-CAL27 and siRNA_EREG_-CAL27, 23 ± 6% and 49 ± 7% inhibition by CTX for siRNA_ctrl_-CAL33 and siRNA_EREG_-CAL33 and 27 ± 5% and 45 ± 5 inhibition by CTX for siRNA_ctrl_-SCC9 and siRNA _EREG_-SCC9, respectively).

Reduced clonogenic survival is associated with an altered growth capacity, reflected here by the inability of cells to reach confluency. Again, CTX is more prone to blocking the growth of cells silenced for EREG (Figure 1C; with 19 ± 1% and 28 ± 1% inhibition by CTX for siRNA_ctrl_-CAL27 and siRNA_EREG_-CAL27, 39 ± 1% and 46 ± 1% inhibition by CTX for siRNA_ctrl_-CAL33 and siRNA_EREG_-CAL33 and 17 ± 3% and 5 ± 2% inhibition by CTX for siRNA_ctrl_-SCC9 and siRNA_EREG_-SCC9, respectively).

The cleavage of PARP, reflecting apoptosis induction, could only barely be detected and only in cells exposed to CTX (Figure 1D). No additional cleavage could be observed in siRNA_EREG_-transfected cells when compared to the controls. Thus, apoptosis induction could not account for the reduction in survival and growth observed in siRNA_EREG_-cells remaining untreated or treated with CTX. Taken together, these data show that the concomitant silencing of EREG and EGFR targeting would be more effective in inhibiting tumor growth and survival.

### 3.2. Silencing EREG Promotes Mitochondrial Dysfunction and Inhibits Autophagy in Reponse to CTX

**Figure 2:**
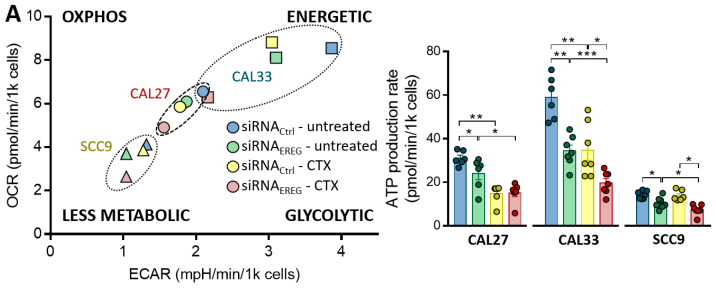

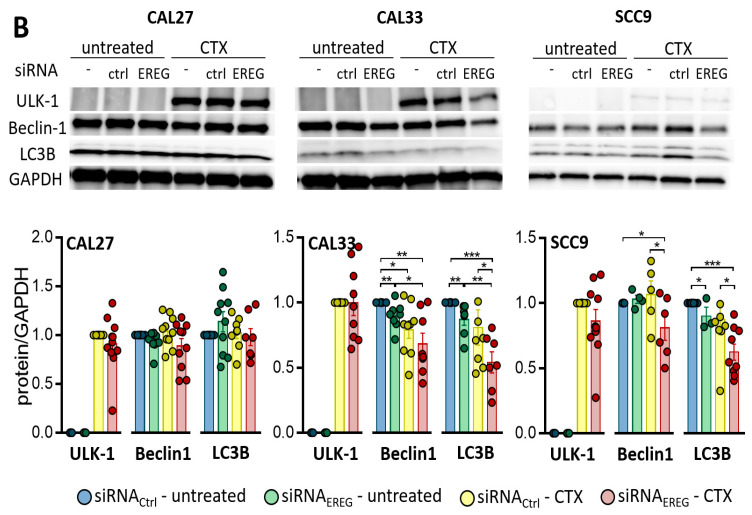
(**A**) Energetic map of CAL27, CAL33 and SCC9 cells transfected with siRNA_Ctrl_ or siRNA _EREG_ and treated with solvent or 30 nM CTX. Histograms show the ATP production of CAL27, CAL33 and SCC9 cells transfected with siRNA_Ctrl_ or siRNA_EREG_ and treated with solvent or 30 nM CTX. Data are represented as the mean ± SEM of ATP produced 144 h post-transfection and post-treatment (*n* = 8–9, with * *p* < 0.05, ** *p* < 0.001 and *** *p* < 0.0001). (**B**) Expression of ULK-1, Beclin1, LC3B and GAPDH was determined by Western blot in CAL27, CAL33 and SCC9 cells not transfected or transfected with siRNA_Ctrl_ or siRNA_EREG_ and treated with solvent or 30 nM CTX. Histograms represent the mean ± SEM (CAL27 *n* = 11, CAL33 *n* = 9 and SCC9 *n* = 10, with * *p* < 0.05, ** *p* < 0.01 and *** *p* < 0.001) of the protein expression normalized with GAPDH.

The metabolic reprogramming of cancer cells has a beneficial effect not only on tumor growth and survival but also on metastasis and chemoresistance. We therefore investigated how EREG might affect the mitochondrial metabolism. The ATP Rate assays reveal that all three cell lines exhibit different basal oxygen consumption (OCR), extracellular acidification (ECAR) and ATP production. CAL33 cells are the most energetic (Figure 2A, left). EREG-silencing significantly reduces OCR and ECAR (Figure 2A, right) and ATP production in all three cell lines, with the highest efficiency in the most energetic cell line, CAL33 (Figure 2A, right, with a 22 ± 3%, 41 ± 3% and 26 ± 1% inhibition in siRNA_EREG_-CAL27, siRNA_EREG_-CAL33 and siRNA_EREG_-SCC9, respectively). Although CTX significantly reduces the production of ATP in both siRNA_ctrl_- and siRNA_EREG_-transfected cells, SCC9 seem less sensitive to it (Figure 2A, right). EREG-silenced cells treated with CTX appear less metabolically active. Thus, silencing EREG and blocking EGFR with CTX cause mitochondria dysfunction, which is more important in highly metabolic cells.

Autophagy is a critical protective mechanism against mitochondrial dysfunction. It maintains cellular homeostasis by removing damaged macromolecules and organelles, including mitochondria. The expression of ULK-1, a kinase regulating the early stages of the autophagosome formation, is only induced in cells exposed to CTX, and no differences are observed between siRNA_ctrl_- and siRNA_EREG_-transfected cells. In contrast, the silencing EREG reduces the expression of Beclin1 and LC3B in CAL33 and the expression of LC3B in SCC9 without affecting CAL27 cells (Figure 2B). Although CTX does not affect Beclin1 and LC3B by itself in any cell line tested, it reduces their expression even further in siRNA_EREG_-transfected CAL33 and SCC9 cells without affecting CAL27 cells (Figure 2B). The data show that silencing both EREG and EGFR signaling inhibits autophagy.

### 3.3. Silencing EREG Promotes Ferroptosis in Response to CTX

**Figure 3:**
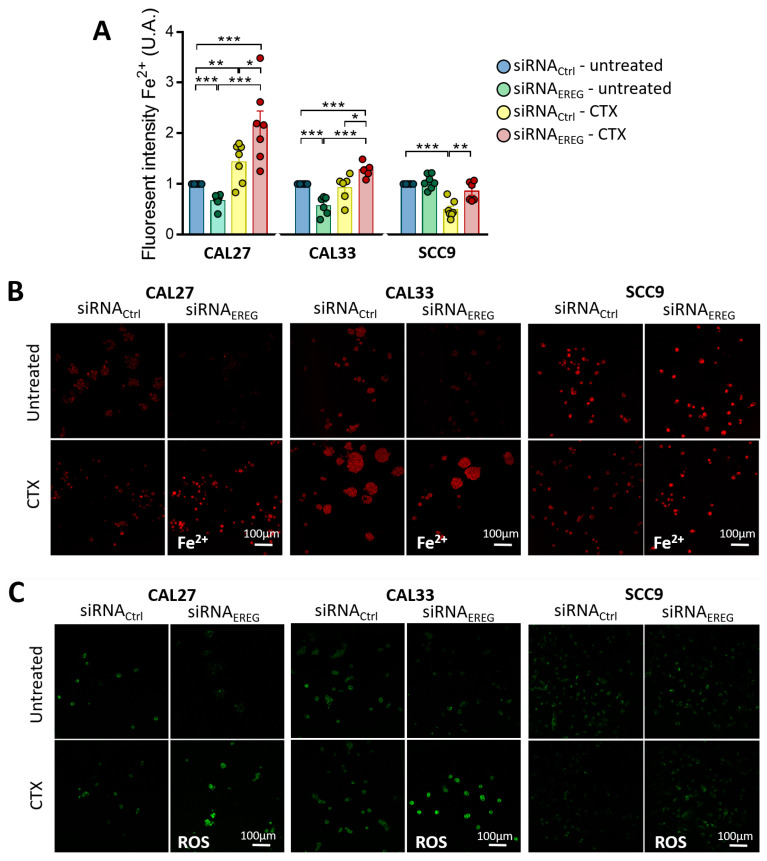
(**A**) Histograms showing the levels of intracellular Fe^2+^ measured in CAL27, CAL33 and SCC9 cells transfected with siRNA_Ctrl_ or siRNA_EREG_ and treated with solvent or 30 nM CTX. Data are represented as the mean ± SEM of intracellular Fe^2+^ intensity (*n* = 8, with * *p* < 0.05, ** *p* < 0.001 and *** *p* < 0.0001). Pictures show (**B**) the intracellular Fe^2+^ production (in red, staining of individual CAL27 and SCC9 cells or clustered CAL33) and (**C**) the lipid peroxidation (in green) acquired by confocal microscopy in CAL27, CAL33 and SCC9 cells transfected with siRNA_Ctrl_ or siRNA_EREG_ and treated with solvent or 30 nM CTX (scale bar: 100 µm).

We next focused on ferroptosis, a different class of cell death, characterized by the accumulation of ferrous ions (Fe^2+^) and the increase in the production of lipid reactive oxygen species (ROS). The accumulation of Fe^2+^ was monitored using the FerroOrange probe. Silencing EREG significantly reduces Fe^2+^ staining in CAL27 and CAL33 cells (Figure 3A,B, with a 33 ± 5%, 43 ± 7% and 0 ± 5% inhibition in siRNA_EREG_-CAL27, siRNA_EREG_-CAL33 and siRNA_EREG_-SCC9, respectively). Although CTX does not affect Fe^2+^ accumulation in siRNA_ctrl_-cells, it significantly induces Fe^2+^ staining in siRNA_EREG_-transfected CAL27 and CAL33 without affecting SCC9 (Figure 3A,B). Lipid peroxides were monitored using the LiperFluo probe and revealed similar staining profiles to the ones observed in Figure 3B. Thus, silencing EREG reduces the accumulation of lipid peroxides in CAL27 and CAL33 cells (Figure 3C). Turning to CTX, it significantly induces lipid peroxides staining in siRNA_EREG_-transfected CAL27 and CAL33 without affecting SCC9 (Figure 3C). Altogether, EREG-silencing reprograms cells to induce ferroptosis in the presence of CTX.

### 3.4. EREG-Silencing Uncovers the Vulnerability of Cells to GPX4 Inhibition

**Figure 4:**
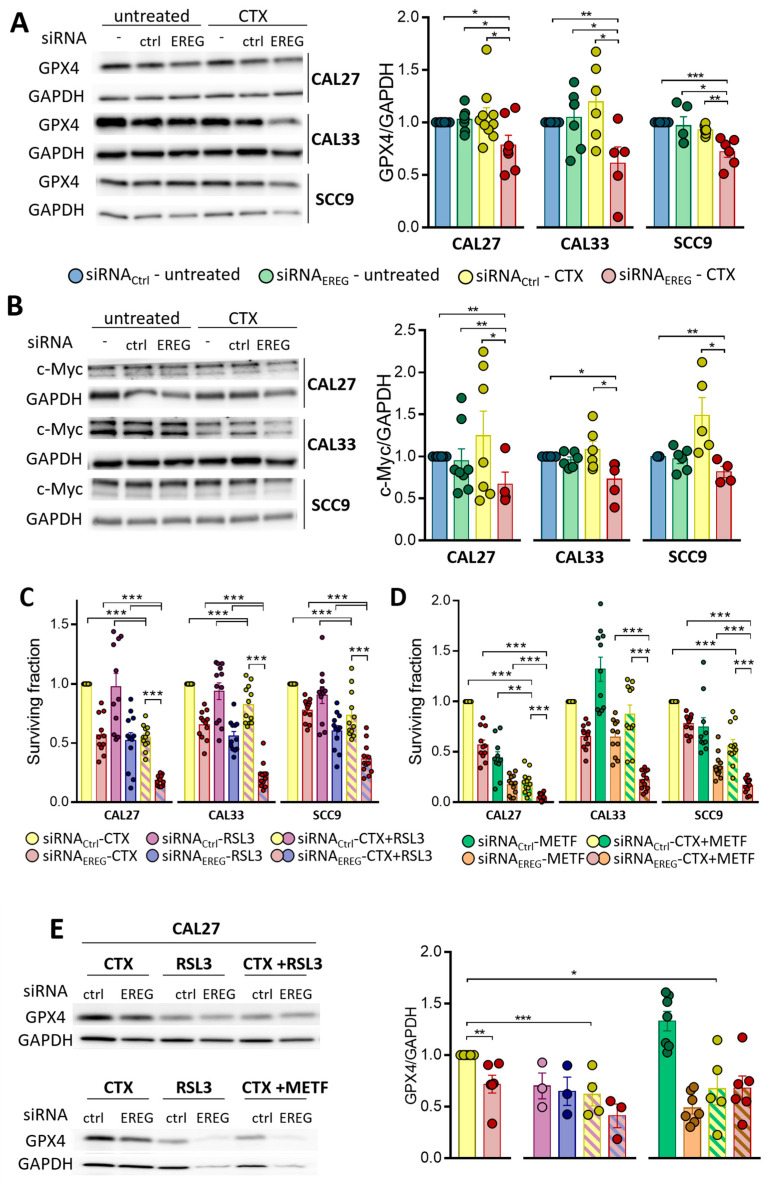
Expression of GPX4 and GAPDH (**A**) and c-Myc and GAPDH (**B**) was determined by Western blot in CAL27, CAL33 and SCC9 cells untransfected or transfected with siRNA_Ctrl_ or siRNA_EREG_ and treated with solvent or 30 nM CTX. Histograms represent the mean ± SEM (CAL27 and CAL33 *n* = 10 and SCC9 *n* = 9, with * *p* < 0.05, ** *p* < 0.01 and *** *p* < 0.001) of the protein expression normalized with GAPDH. (**C**) Histograms show the surviving fraction of CAL27, CAL33 and SCC9 cells transfected with siRNA_Ctrl_ or siRNA_EREG_ and treated with 30 nM CTX, 5 µM RSL3 or a combination of both. Data represent the mean ± SEM surviving fraction at day 8 post-transfection and post-treatment (*n* = 4, with *** *p* < 0.0001). (**D**) Histograms show the surviving fraction of CAL27, CAL33 and SCC9 cells transfected with siRNA_Ctrl_ or siRNA_EREG_ and treated with 30 nM CTX, 1 mM metformin (METF) or a combination of both. Data represent the mean ± SEM surviving fraction at day 8 post-transfection and post-treatment (*n* = 4, with ** *p* < 0.01 and *** *p* < 0.0001). (**E**) Expression of GPX4 and GAPDH was determined by Western blot in CAL27 transfected with siRNA_Ctrl_ or siRNA_EREG_ and treated with 30 nM CTX, 5 µM RSL3 or a combination of both (upper panel) or with 30 nM CTX, 1 mM metformin (METF) or a combination of both (lower panel). Histograms represent the mean ± SEM (*n* = 4, with * *p* < 0.05, ** *p* < 0.01 and *** *p* < 0.001) of the protein expression normalized with GAPDH.

It has been shown that ferroptosis is initiated either by the loss of glutathione peroxidase 4 (GPX4, an enzyme involved in lipid repair) or the depletion of cystine. GPX4, together with its co-factor glutathione (GSH), catalyzes the inhibition of lipid peroxides. Its loss is concomitant with the accumulation of lipid peroxides in membranes, which leads to ferroptosis. Silencing EREG does not affect GPX4 expression in any of the cell lines tested. The exposure of siRNA_ctrl_-transfected cells to CTX does not affect it either (Figure 4A). However, the treatment of siRNA_EREG_ cells with CTX results in a significant inhibition of GPX4 expression in all three cell lines (Figure 4A; with a 24 ± 9%, 42 ± 6% and 26 ± 5% inhibition in siRNA_EREG_-CAL27, siRNA_EREG_-CAL33 and siRNA_EREG_-SCC9, respectively).

The cystine/glutamate antiporter system (also called x-c or xCT (coded by the genes SLC7A11 and SLC3A2)) imports extracellular cystine that will be further reduced into cysteine. Cysteine acts as a precursor for the synthesis of GSH, the cofactor of GPX4. GPX4 is also a direct transcriptional target of NRF2. SLC7A11 (as well as SLC1A5 and SLC7A5) and NRF2 are under the control of the oncogene c-Myc [23]; we investigated how EREG and/or CTX might affect c-Myc expression in our system. Silencing EREG or exposing siRNA_ctrl_-transfected cells to CTX does not affect c-Myc expression in any of the cell lines tested (Figure 4B). However, the treatment of siRNA_EREG_ cells with CTX results in a significant inhibition of c-Myc expression in all three cell lines (Figure 4B; with a 33 ± 14%, 23 ± 9% and 19 ± 6% inhibition in siRNA_EREG_-CAL27, siRNA_EREG_-CAL33 and siRNA_EREG_-SCC9, respectively).

Pharmacological inhibitors such as RSL3 have been reported to either degrade GPX4 or inhibit its function. RSL3 reduces the surviving fraction of siRNA_ctrl_- and siRNA_EREG_-transfected cells to similar levels as CTX (Figure 4C compared to 1B for untreated cells). The surviving fraction of both siRNA_ctrl_- and siRNA_EREG_-transfected cells is even further inhibited when RSL3 is combined with CTX (Figure 4C). Metformin, already used in the treatment of diabetes, was recently described as promoting ferroptosis in different ways, including by the inhibition of SLC7A11 [24,25]. We therefore studied the effects of this non-specific inducer of ferroptosis in our model. Metformin reduces the surviving fraction of siRNA_ctrl_- and siRNA_EREG_-transfected cells to similar levels as CTX in CAL33, but it was far more potent in CAL27 and SCC9 cells (Figure 4D, compared to 1B for untreated cells). The surviving fraction of both siRNA_ctrl_- and siRNA_EREG_-transfected cells is even further inhibited when metformin is combined with CTX (Figure 4D). The exposure of the cells to RSL3 or metformin alone or in combination with CTX results in a significant inhibition of GPX4 expression, which is even more pronounced in EREG-silenced cells (Figure 4E). Altogether, the data show that GPX4 is crucial for cell survival and that its disappearance sensitizes to CTX.

### 3.5. GPX4 Inhibition Sensitizes the Patient-Derived Tumoroid to CTX

**Figure 5:**
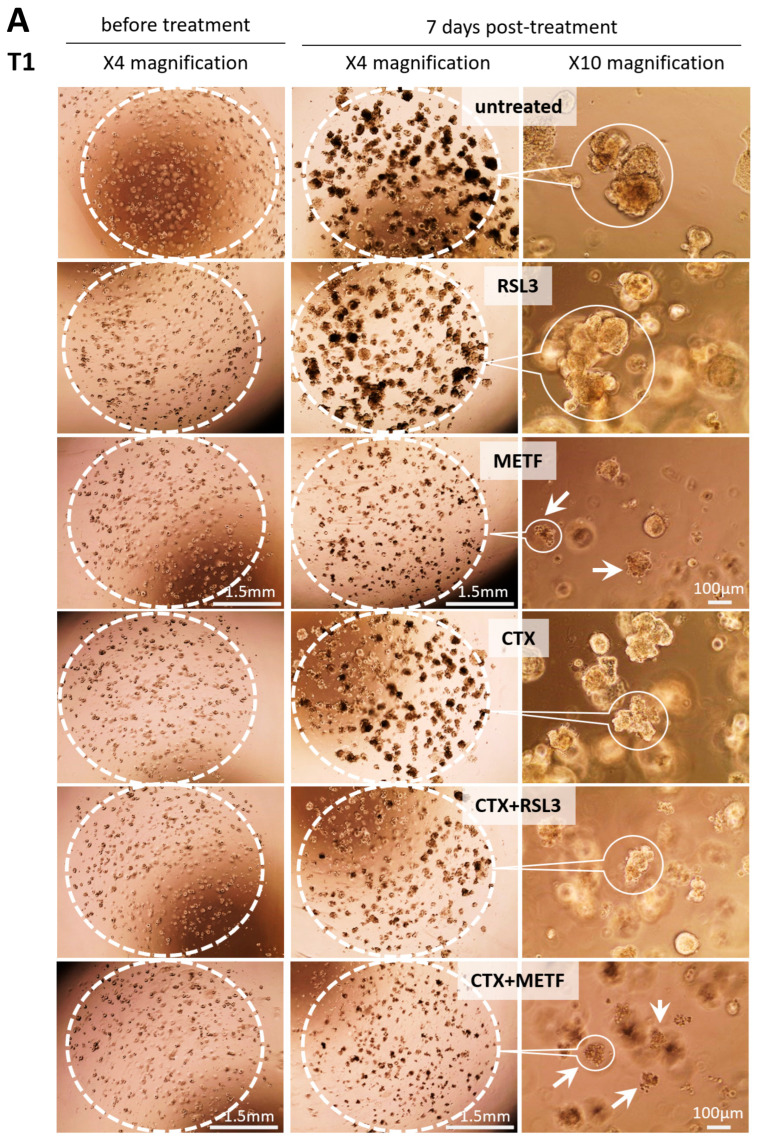

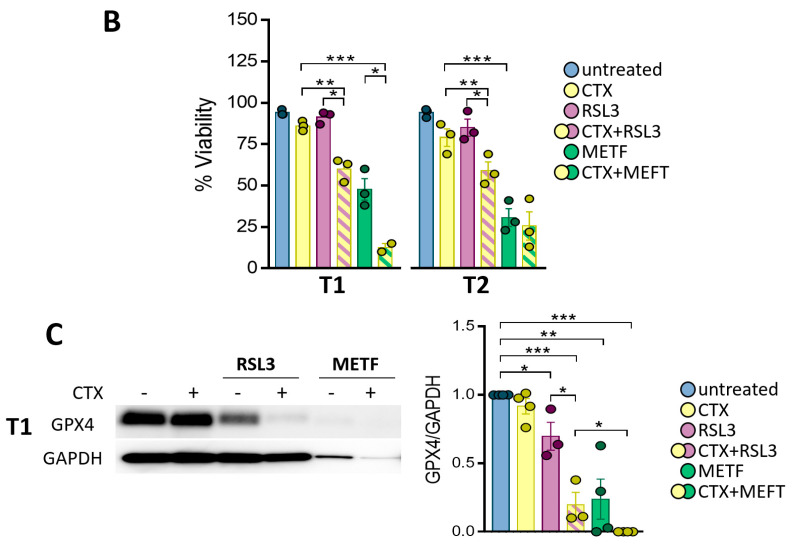
(**A**) Patient-derived tumoroids T1 were treated with solvent, 30 nM CTX, 5 µM RSL3, 1 µM metformin (METF), a combination of CTX + RSL3 or CTX + metformin. Pictures were before treatment (left panel) and 7 days after treatment with 4× (middle panel) and 10× (right panel) magnification. (**B**) Viability was determined after 7 days of the culture. Each bar represents the mean ± SEM of the percentage of viability (*n* = 3 for T1 and T2, with * *p* < 0.05, ** *p* < 0.001, *** *p* < 0.0001. (**C**) Expression of GPX4 and GAPDH was determined by Western blot in T1 tumoroids treated with solvent, 30 nM CTX, 5 µM RSL3, 1 µM metformin (METF), a combination of CTX + RSL3 or CTX + metformin. Histograms represent the mean ± SEM (*n* = 4, with * *p* < 0.05, ** *p* < 0.01 and *** *p* < 0.001) of the protein expression normalized with GAPDH.

In order to validate our hypothesis, we exposed patient-derived tumoroids to CTX, RSL3, metformin and a combination of drugs for 7 days. Tumoroids are treated 7 days after plating in 3D drops of basement membrane extract (BME dotted circle, Figure 5A, left panel before treatment) to allow for formation. After 7 days of treatment, the tumoroids were photographed (pictures only shown for T1) at a low magnification (×4, Figure 5A middle panel) to follow the growth in the 3D BME drops (dotted circle) characterized by an increase in the size and at a high magnification (×10, Figure 5A right panel) to observe the variations in the morphology related to the different treatments. CTX and RSL3 alone do not affect the growth or the viability (Figure 5A,B) of tumoroids when compared to the untreated tumoroids. In contrast, the combination of CTX and RSL3 clearly reduces the size (Figure 5A) and the viability to 60 ± 4% and 59 ± 5% in tumoroids 1 and 2, respectively (Figure 5B). The non-specific inducer of ferroptosis, metformin by itself, reduces the size (Figure 5A) and the viability of tumoroids 1 and 2 to 48±6% and 31 ± 5%, respectively (Figure 5B). The combination of CTX and metformin reduces the viability even further in tumoroid 1 but not in tumoroid 2 (12 ± 2% and 25 ± 8% in tumoroids 1 and 2, respectively; Figure 5B) and is more efficient than CTX and RSL3. Reduced viability is associated with the appearance of debris in 3D BME drops (arrows in Figure 5A).

Finally, the exposure of tumoroids to CTX does not affect GPX4 expression (Figure 5C). GPX4 is significantly reduced by RSL3 alone (30 ± 10%) and even further when RSL3 is combined with CTX (80 ± 9%). Similar results were obtained in T2 and T3 (data not shown). It is also significantly reduced by metformin alone (76 ± 14%) and totally lost when metformin is combined with CTX (Figure 5C). Altogether, the data confirm the efficacy of targeting xCT and GPX4 in CTX-resistant tumors.

## 4. Discussion

The dysregulation of EREG may contribute to the progression of various cancers including HNSCC and may be a putative mechanism of resistance to EGFR-targeted therapies. EREG is usually overexpressed in HNSCC and correlates with short overall survival and progression-free survival [11,15,18,19,20]. EREG conducts an even more potent mitogenic signal than EGF in HNSCC mimicking EGFR oncogenic mutations [11,20]. Job et al. recently described a subgroup of HPV-negative HNSCC named “basal” sharing molecular similarities such as the upregulation of genes involved in the EGFR signaling pathway including EREG and AREG [21]. Cells sharing these characteristics appear to be more sensitive to EGFR-targeted therapy, with CTX being the least efficient. Because the suppression of EREG expression reduces cell survival, the authors suggested that cells may be addicted to an EREG feedback loop and that EREG should be considered as a functional biomarker for HNSCC sensitivity to EGFR blockade [21]. In line with this study, we observed that HNSCC tumor cells expressing caveolin-1 could use EREG silencing, but not AREG silencing, to overcome oncogenic dependence on EGFR and develop resistance to CTX/irradiation combination therapy [8]. The resistance was due, at least in part, to the silencing of the HIPPO pathway, leading to the activation of YAP/TAZ [8]. The cross-suppression of both AREG and EREG has also been reported to lead to the emergence of CTX resistance, which is related to the loss of cell addiction to EGFR, compensated by the hyperactivation and addiction to FGFR3 in melanoma [26]. We show here that the direct suppression of EREG expression reduces both EGFR expression and HNSCC basal cell survival. Rather than driving resistance to CTX, the loss of EREG reduces survival even further. While it cannot be excluded that long-term EREG silencing may lead to the emergence of CTX resistance, the acute targeting of EREG combined with CTX is effective in reducing cell survival and could be a feasible antitumor strategy for HNSCC. Fepixnebart (LY3016859, developed by Eli Lilly and Co.) is a monoclonal antibody that binds epiregulin and TGF-α and is well tolerated and efficient in neutralizing both targets [27]. It is currently in phase II for back pain and neuropathic pain and in phase III for diabetic neuropathies. It would be interesting to determine its anti-tumor effect and adjuvant effect for EGFR-targeting therapies. Besides its autocrine feedback loop, EREG is also secreted by the component of the tumor microenvironment such as macrophages [15] and cancer-associated fibroblasts (CAF) [28]. Macrophages-derived EREG induces EGFR-TKI resistance in NSCLC, and CAF-derived EREG promotes OSCC invasion and metastasis through the induction of EMT. Thus, targeting EREG might not only prevent therapy resistance but also HNSCC progression.

Aberrant metabolism and metabolism reprogramming represent malignant tumor hallmarks that are required for cancer cells to proliferate and progress. The metabolism of cancer cells is mainly based on nonoxidative glycolysis, followed by the fermentation of lactic acid to produce ATP, a phenomenon known as the Warburg effect. EREG/EGFR signaling enhances glycolysis by increasing glucose consumption, lactate production, extracellular acidification (ECAR) and the intracellular levels of ATP as well as by activating several glycolytic genes [29,30]. However, HNSCC also depend on glutamine for producing energy [31], which is imported in cells by the Na+-glutamine/Na+-cysteine exchanger ASCT2. Besides serving as a source of carbon and nitrogen for macromolecule synthesis, glutamine provides α-ketoglutarate for the tricarboxylic acid (TCA) cycle and contributes to the production of the most powerful antioxidant, glutathione (GSH) (for a review, see [23]). The production of GSH also requires cysteine, which is imported into cells through the x-c or xCT cystine/glutamate antiporter. GSH serves as a cofactor of the glutathione peroxidase 4 (GPX4) to suppress destructive lipid reactive oxygen species (ROS). This pathway plays a key role in the regulation of ferroptosis, which is a regulated cell death triggered by an iron-dependent lipid peroxidation [32]. Both GPX4 and x-c antiporter are crucial regulators of ferroptosis. Here, we show that silencing EREG as well as blocking EGFR lead to mitochondrial defects characterized by a reduction in ATP production, oxygen consumption (OCR) and ECAR. Combining EREG silencing with an EGFR blockade shifts cells from an energetic state toward a less metabolic phenotype. If the dysfunction of the mitochondria could affect cell survival through energetic stress, death could neither be attributed to apoptosis, which was undetectable, or to autophagy, which was inhibited. Mitochondria play a major role in regulating oxidative metabolism, are the main source of reactive oxygen species (ROS) and are the primary site of Fe^2+^ iron storage and utilization. Therefore, the dysregulation of mitochondria induced by the silencing of EREG and the inhibition of EGFR might alter the iron metabolism and generate a redox imbalance strong enough to trigger ferroptosis. Here, we show that the silencing of EREG combined with the blockade of EGFR lead to the accumulation of Fe^2+^ and lipid peroxides associated with the downregulation of GPX4. No ferroptosis could be achieved by the loss of EREG alone or by the treatment with CTX. Accordingly, although CTX downregulates ASCT2 via a CTX-dependent EGFR endocytosis, it does not alter survival by itself. However, it decreases the intracellular uptake of glutamine and the levels of GSH, which sensitizes HNSCC to ROS-induced death [33,34]. In colorectal cancer cells, CTX neither affected proliferation or survival by itself, even though it inhibited NRF2 signaling (known to promote GPX4 and HO-1 transcription). By targeting NRF2/HO-1, CTX enhances RSL-3-induced ferroptosis [35]. Further studies will be needed to determine if those molecular targets are also altered by the loss of EREG. However, we did show the inhibition of c-Myc and GPX4 expression in EREG-silenced cells treated by CTX. We previously reported that the oncogene c-Myc, a known target of EREG/EGFR, is downregulated by CTX in EREG-/caveolin-1-expressing cells [8,11]. As c-Myc regulates ASCT2, LAT1, x-c antiporter as well as NRF2 [23,36], it deserves further investigations.

Inducing ferroptosis seems to be an attractive potential anti-cancer strategy with broad clinical implications. Several preclinical studies show that ferroptosis inducers can synergize with traditional chemotherapeutics [35,37,38]. They either target the depletion of the cellular antioxidant GSH through the x-c antiporter (Erastin) or directly target GPX4 (RSL3). Here, the loss of GPX4 is associated with the induction of ferroptosis in EREG-silenced cells exposed to CTX. Inhibiting GPX4 by using RSL3 reduces the survival of control cells exposed to CTX to levels equivalent to those observed in EREG-silenced cells exposed to CTX. However, RSL3 also sensitizes EREG-silenced cells to CTX to an even greater extent. The maximum decrease in cell viability corresponds to a steep decrease in GPX4. The data uncover the value of targeting GPX4 to effectively sensitize tumor cells to CTX. Accordingly, the overexpression of GPX4 was described in EGFR-TKI-resistant lung adenocarcinoma and colorectal cancers. RSL3 restores their sensitivity to EGFR-TKI [35,38]. Similar results could be obtained using metformin, which is widely used for the treatment of type 2 diabetes mellitus (T2DM). Its use in T2DM has been associated with cancer incidence and mortality decreases, including in HNSCC (for a review, see [39,40,41,42]). This effect seems to be due to the reduction in circulating insulin, since both the insulin–IGF system and hyperglycemia have been associated with cancer risk. However, metformin also has a direct anti-tumor effect via the induction of energetic stress. It inhibits the mitochondrial respiratory chain complex I, leading to mitochondrial dysfunctions, changes in the levels of ROS and the iron homeostasis (for a review, see [43]). Acting independently of GPX4, metformin also downregulates SLC7A11 (the catalytic unit of the x-c cytine/glutamate antiporter), protein stability and expression by inhibiting UFM1 expression and the subsequent UFMylation of SLC7A11 [25]. Metformin increases intracellular total ROS and lipid ROS levels and reduces intracellular GSH, which ultimately leads to ferroptosis [25]. Metformin was also recently reported to induce ferroptosis in breast cancer by inhibiting autophagy [44]. In our hands, metformin was more effective in reducing cell survival than RSL3, which is probably due to its multiple targets. Its co-administration with CTX in control cells demonstrated an adjuvant effect of metformin that is even more pronounced in EREG-silenced cells. Again, the decrease in cell viability is associated with the loss of GPX4. Although the targeting of the x-c antiporter or GPX4 sensitizes control cells to CTX treatment, the most striking effects are seen in cells where EREG is lost.

Tumoroids are 3D tumor-resembling cellular clusters generated from primary patient material. They closely recapitulate the 3D tissue architecture, cellular composition and characteristics (including genetic and cellular intratumor heterogeneity as well as resistance to therapy) of the tumor from which they were derived, offering useful benefits over conventional 2D cell culture and 3D multicellular spheroids. They can be grown long-term without genetic or functional changes [45,46]. The results obtained to date indicate that tumoroids respond in a largely consistent manner to the patients they were derived from [47], show heterogeneous sensitivities to standard treatments [48,49] and might predict a patient’s clinical outcome. Tumors hold promise for biomarker identification, drug discovery and aiding personalized therapy. For all these reasons, we have chosen to generate HNSCC tumoroids to validate our therapeutic approaches combining ferroptosis inducers with CTX. As a proof of concept, patient-derived HNSCC tumoroids showing resistance to CTX were co-treated with RSL3 or metformin. Tumoroids survival was drastically decreased with RSL3-CTX co-treatment and was almost completely abrogated in response to metformin-CTX. Metformin combined with CTX was therefore more effective in reducing viability than RSL3/CTX. As stated above, it might be related to the fact that RSL3 only targets GPX4, whereas metformin has a multitude of targets, some of which, such as SCL7A11, act further upstream in antioxidant signaling. The data also underline a heterogeneity in the response of tumoroids. Indeed, if metformin sensitizes tumoroid 1 to CTX, this is not the case for the second. An inhibition of EGFR expression by metformin could lead to this desensitization to CTX, as previously observed [50]. Further studies will be necessary to understand this heterogeneity. The maximum diminution of cell viability is associated with the strongest reduction in GPX4 levels.

## 5. Conclusions

To our knowledge, this is the first study reporting that a loss of EREG might sensitize HNSCC to CTX through the induction of ferroptosis. To date, only a high expression of EREG was considered to predict the response of a patient to anti-EGFR therapies. However, care should be taken, since emerging studies report that secreted EREG in the microenvironment might support therapy resistance and tumor progression [15,28]. Thus, using EREG expression levels to identify patients likely to benefit from EGFR-TKI therapies could lead to the exclusion of some who would be better responders. Here, we propose combining ferroptosis inducers with CTX. Our data clearly show that the combination of both reduces the survival of tumors expressing EREG and that the effect is even more pronounced in tumors where EREG is lost. This is also the first study validating the efficacy of using ferroptosis inducers in combination with CTX to inhibit survival in a patient-derived tumoroid model resistant to CTX. In conclusion, a loss of EREG might be considered in clinical settings as a predictive biomarker for patients that might benefit the most from the combination of ferroptosis inducers and CTX.

## Data Availability

Not applicable.

## Supplementary Materials

The following supporting information can be downloaded at: https://www.mdpi.com/article/10.3390/cells12050733/s1, Table S1: Antibodies.
