# Supplementary material for: Blocking EREG/GPX4 Sensitizes Head and Neck Cancer to Cetuximab through Ferroptosis Induction
Source: Cells. 2023 Feb 24;12(5):733. doi: 10.3390/cells12050733 (PMC10000618; doi:10.3390/cells12050733)
Supplement: Supplementary file 1 [file cells-12-00733-s001.zip › cells-2074315-supplementary.pdf]

Table S1. Antibodies.

Antibody	Species	Dilution	Suppliers
Beclin-1 #3738	Rabbit	1:1000	Cell Signaling Technology©
Caveolin-1 (D4663) #3267S	Rabbit	1:1000	
EREG (D4O5I) #12048	Rabbit	1:1000	
EGFR (D38B1) #4267S	Rabbit	1:1000	
LC3B (D11) #3868	Rabbit	1:1000	
Cleaved PARP (Asp214) #9541	Rabbit	1:1000	
ULK1 (D8H5) #8054	Rabbit	1:1000	
GAPDH (MAB374)	Mouse	1:10000	EMD Millipore©
GPX4 (EPNCIR144) #ab125066	Rabbit	1:1000	Abcam©
c-MYC (9E10) sc-40	Mouse	1:200	Santa Cruz Biotechnology©
Anti-mouse IgG HRP-conjugate	Goat	1:10000	Promega©
Anti-rabbit IgG HRP-conjugate			
